# Quality of care for tuberculosis and HIV in the private health sector: a cross-sectional, standardised patient study in South Africa

**DOI:** 10.1136/bmjgh-2021-005250

**Published:** 2021-05-14

**Authors:** Jody Boffa, Sizulu Moyo, Jeremiah Chikovore, Angela Salomon, Benjamin Daniels, Ada T Kwan, Madhukar Pai, Amrita Daftary

**Affiliations:** 1 Dahdaleh Institute of Global Health Research, York University, Toronto, Ontario, Canada; 2 Centre for Rural Health, University of KwaZulu-Natal, Durban, South Africa; 3 Human and Social Capabilities Division, Human Sciences Research Council, Cape Town, South Africa; 4 School of Public Health, University of Cape Town, Cape Town, South Africa; 5 Human and Social Capabilities Division, Human Sciences Research Council, Durban, South Africa; 6 McGill International TB Centre, McGill University, Montreal, Québec, Canada; 7 International Public Health, Georgetown University, Washington, DC, USA; 8 Department of Medicine, University of California San Francisco, San Francisco, California, USA; 9 School of Global Health, York University, Toronto, Ontario, Canada; 10 Centre for the AIDS Programme of Research in South Africa, University of KwaZulu-Natal, Durban, South Africa

**Keywords:** tuberculosis, HIV, health services research, cross-sectional survey, epidemiology

## Abstract

**Background:**

South Africa has high burdens of tuberculosis (TB) and TB-HIV, yet the quality of patient care in the private sector is unknown. We describe quality of TB and TB-HIV care among private general practitioners (GPs) in two South African cities using standardised patients (SPs).

**Methods:**

Sixteen SPs presented one of three cases during unannounced visits to private GPs in selected high-TB burden communities in Durban and Cape Town: case 1, typical TB symptoms, HIV-positive; case 2, TB-specified laboratory report, HIV-negative and case 3, history of incomplete TB treatment, HIV-positive. Clinical practices were recorded in standardised exit interviews. Ideal management was defined as relevant testing or public sector referral for any reason. The difference between knowledge and practice (know-do gap) was assessed through case 1 vignettes among 25% of GPs. Factors associated with ideal management were assessed using bivariate logistic regression.

**Results:**

511 SP visits were completed with 212 GPs. Respectively, TB and HIV were ideally managed in 43% (95% CI 36% to 50%) and 41% (95% CI 34% to 48%) of case 1, 85% (95% CI 78% to 90%) and 61% (95% CI 73% to 86%) of case 2 and 69% (95% CI 61% to 76%) and 80% (95% CI 52% to 68%) of case 3 presentations. HIV status was queried in 35% (95% CI 31% to 39%) of visits, least with case 1 (24%, 95% CI 18% to 30%). The difference between knowledge and practice was 80% versus 43% for TB and 55% versus 37% for HIV, resulting in know-do gaps of 37% (95% CI 19% to 55%) and 18% (95% CI −1% to 38%), respectively. Ideal TB management was associated with longer visit time (OR=1.1, 95% CI 1.1 to 1.2), female GPs (3.2, 95% CI 2.0 to 5.1), basic symptom inquiry (2.0, 95% CI 1.7 to 2.3), HIV-status inquiry (OR=11.2, 95% CI 6.4 to 19.6), fewer medications dispensed (OR=0.6, 95% CI 0.5 to 0.7) and Cape Town (OR=2.2, 95% CI 1.5 to 3.1). Similar associations were observed for HIV.

**Conclusions:**

Private providers ideally managed TB more often when a diagnosis or history of TB was implied or provided. Management of HIV in the context of TB was less than optimal.

**Key questions**

**What is already known?**

- South Africa ranks among the highest tuberculosis (TB) and HIV-associated TB burden countries globally and also has a thriving private healthcare sector.
- Studies using standardised patients (SPs) to evaluate private sector TB management globally have demonstrated generally poor quality of care (range 12%–38% ideal management).
- No SP studies have evaluated private sector TB management in South Africa or HIV management in the context of TB globally.

**What are the new findings?**

- When presented with an SP reporting typical TB symptoms and HIV on probing, private general practitioners (GPs) managed TB and HIV ideally (sent for appropriate tests or referred to the public sector) in 43% and 41% of visits, respectively.
- Comparatively, GPs performed better when SPs presented with TB symptoms and a confirmatory laboratory report (HIV-negative on probing): 85% and 61% for TB and HIV, respectively, or with a history of incomplete TB treatment (HIV-positive on probing): 69% and 80% for TB and HIV, respectively.
- Ninety per cent of ideal management involved referral to the public sector, yet only a third of GPs inquired directly about HIV status.
- GPs managed TB 37% more often and HIV 18% more often in case 1 vignettes compared to SP visits. GP concerns about patients’ ability to afford or access appropriate TB tests through public or private means may explain the know-do gap.

**Key questions**

**What do the new findings imply?**

- Private GPs in South Africa performed as well or better than private providers in similar studies in India and Kenya, but less well compared to the public sector.
- Strategies that support linkage to affordable testing may improve management practices when TB is considered as a differential diagnosis.
- Further research is needed to identify barriers to querying HIV status in this high HIV prevalence setting.

## Introduction

Tuberculosis (TB) remains the world’s leading cause of death from a single infectious agent.^1^ With over 300 000 new cases each year, South Africa has the third highest incidence of TB globally, and 59% of people with TB have HIV-coinfection.^1^ However, clinical outcomes for TB and HIV-associated TB are suboptimal. Only 54% of patients successfully navigate the care cascade, from symptom presentation to treatment completion.^2^ Delays in TB testing and treatment may lead to increased TB transmission, morbidity and mortality.

The South African health system has been described as a stratified system, with a private subsystem in which a minority of wealthier people access what is generally thought to be higher-quality care, while the poorer generally rely on the lower-tiered public sector free of user fees.^3^ Primary care clinics in the public system are normally staffed by nursing professionals (and other support staff) whose role includes TB and HIV testing, treatment initiation and monitoring, with referral to medical officers and specialist physicians at community health centres, as appropriate. The first point of contact for primary care in the private sector is most often qualified general practitioners (GPs) who are regulated by the Health Practitioners Council of South Africa. Roughly 20% of South Africans have private medical insurance which commonly covers catastrophic illness with out-of-pocket fees for ambulatory care in the private sector.^3 4^

Yet, patient pathways studies in high-TB burden countries reveal that up to 60% of people with TB begin their care in the private sector.^5^ In South Africa, up to 38% of people are estimated to seek primary care from the private sector, where 37% of the country’s GPs practice.^4^ Patient movement between clinics and health sectors may, among other factors, contribute to losses from the TB care cascade.^6^ A global meta-analysis comparing patient outcomes between public and private sectors suggests that people with TB who access the private sector are less likely to complete TB treatment.^7^ However, clinical practices for TB and TB-HIV have not been well studied in South Africa’s private health sector.^8^

Standardised patient (SP) studies, whereby healthy people trained to portray particular health conditions in a consistent manner visit healthcare facilities as ‘mystery’ patients, have been increasingly used to assess the quality of TB care in both public and private sectors.^9^ The SP method reduces measurement bias compared with vignettes, case presentations or clinical observations in which doctors are aware of being evaluated.^9^ An SP study in South Africa’s public sector reported that 81% of nurses offered TB tests and 49% offered HIV tests to SPs presenting with typical TB symptoms.^10^ In the private sector, updates on TB guidelines are typically made available through optional continuing medical education (CME) opportunities such as medical association conferences and sessions offered through independent practice associations. SP studies in the private sector in other settings have found the quality of TB care to be comparatively less than the public sector^11–13^; however, no such studies have been conducted in South Africa’s private sector. To date, there have been no published SP studies looking at HIV management in conjunction with TB symptom presentation in the private sector.

We, therefore, undertook an SP study to describe the quality of TB and HIV-associated TB care and quantify the know-do gaps—the difference between GPs’ intended and observed practices^11^—among private GPs in two urban areas. We hypothesised that private sector GPs would perform less well than clinicians in South Africa’s public sector, yet better than private sector performance in other settings given the burden of disease and strong governmental commitment to addressing TB and HIV nationally. We expected that HIV management would be as good as TB management given the extraordinarily high rates of TB-HIV coinfection in the country.

## Methods

### Design

Using a cross-sectional design, SPs were employed to make unannounced visits to GPs, recalling all management practices through a facilitated exit interview immediately following each visit. Visits were neither audio nor video recorded. Exit interview recall was part of the SP training process. In each city, eight SPs received extensive training in one of three standardised cases (table 1): Four SPs were trained to present case 1, a 33-year-old with ‘typical TB symptoms’ (cough, fever, weight loss, night sweats) living with HIV and not on antiretroviral therapy (ART); two were trained to present case 2, a 25-year-old with a laboratory report specifying TB detection by GeneXpert (‘confirmed TB’), and not known to have HIV and two were trained to present case 3, a 38-year-old with typical TB symptoms, known HIV (no ART) and a history of incomplete TB treatment (‘previous TB’). SP cases appeared to come from low or middle-income backgrounds so as to fit with the profile of patients seen in each practice. Cases were pilot tested with practicing GPs. SPs were also trained and tested in risk mitigation (eg, how to avoid blood tests) and research ethics. All SPs were 25–40 years old, black African and fluent in isiZulu or isiXhosa to match typical case presentations in the respective practices. Of 16 SPs, 11 were women and five were men. All SPs were in apparent good health to minimise confounding.

**Table 1:** Standardised patient case descriptions

Standardised patient case	Opening statement	Relevant history	Ideal TB management strategy	Ideal HIV management strategy
Case 1: typical symptoms	‘I have a cough and am feeling hot, and it’s not getting better’	Cough duration 2 weeks, experiencing loss of weight/appetite and night sweats, known HIV+, not on ART	Offered/sent for any TB test or referred to public sector for any reason	Offered/sent for tests to assess CD4 cell count and/or viral load or referred to public sector for any reason
Case 2: confirmed TB	‘I have a cough that is not getting better. I have been to a clinic back home and they gave me some tablets and took my spit’	Carrying GeneXpert pos/Rif inconclusive laboratory report. Cough duration 3 weeks, experiencing loss of weight/appetite and night sweats, HIV− at last test 1 year ago	Offered/sent for any TB test or referred to public sector for any reason	Offered/sent for rapid or ELISA test for HIV or referred to public sector for any reason
Case 3: previous TB	‘I am suffering from a bad cough. About a year ago I had got tablets in the hospital, and it had got better. But now again I’m having this cough’	Cough duration 2 weeks, experiencing loss of weight/appetite and night sweats, diagnosed and treated with TB last year at which time took 3–4 months TB treatment, known HIV+, not on ART	Offered/sent for any TB test or referred to public sector for any reason	Offered/sent for tests to assess CD4 cell count and/or viral load or referred to public sector for any reason

ART, antiretroviral therapy; TB, tuberculosis.

### Participants

The study setting included urban and peri-urban wards in Durban and Cape Town that met the following criteria: (1) ≥20% of ward with annual household income <ZAR40 000 (approximately US$3000), (2) ≥1000 black Africans by subplace (to minimise SP detection) and (3) presence of >2 private GPs.^14^ Eligible GPs had to be registered with the Health Professions Council of South Africa and practicing in a study community, as confirmed by online listings (eg, Discovery insurance, Medpages database) or community drive-throughs conducted by research staff during the recruitment process. The latter entailed the research team physically surveying each community to capture all private GPs for sampling purposes, including those GPs who may have moved locations or were not listed in online directories. We excluded GPs practicing only at private hospitals a priori, as often many GPs would share a waiting room and not work set hours, making it difficult to organise covert visits. We also excluded GPs catering to specialised populations (eg, paediatrics, pregnant women), as SPs would not fit the expected patient profile. Providers were approached by research staff at their practices to seek informed consent. Providers were informed that they would receive up to three unannounced SPs with undisclosed symptoms over a 6-month period. Additionally, providers were requested to document details of any visit in which they suspected a patient may be an SP (including dates, age, sex and presenting complaint of patient) to determine detection rates in a follow-up survey. GPs were also consented to complete an intake survey and participate in a knowledge survey including a case vignette, if selected.

### Data collection

Immediately following consent, GPs completed a facilitated intake survey to record information about their clinical practice. In each city, between 1 and 5 months after consent, SPs made unannounced visits to GPs.

All GPs were scheduled to receive case 1 (typical TB presentation). In Durban, we randomly selected 50 GPs to receive case 2 (confirmed TB) and 75 to receive case 3 (previous TB). In Cape Town, we increased the proportion of confirmed and previous TB cases assigned to GPs, given available resources. To avoid priming for TB, GPs were scheduled to receive case 1 first, followed by case 3 and/or case 2, depending on random assignment and with a minimum of 2 weeks gap between them.^9^ The same case was not presented to a GP more than once. SPs paid usual cash rates for walk-in consultations and stepped aside if any person was seen to be requiring urgent care. Immediately following a visit, SPs completed a facilitated exit interview at a private location to record observed GP practices. Within 3 months of all SP visits being completed, 25% of GPs sampled (n=50) were randomly selected to participate in a facilitated knowledge survey, comprising a case vignette (which mimicked case 1), followed by open-ended and closed general knowledge questions about symptoms, diagnosis and clinical decision-making for TB and HIV. All GPs were followed-up to complete a telephone-based detection survey within 4 months of completed visits.

### Analysis

All survey data were checked for completion, entered into SurveyCTO and cross-checked with the original data to ensure accuracy. The South African TB guidelines^15^ were used as a reference for ideal TB and HIV management, which was defined as a verbal or written (1) recommendation for any TB or HIV-related test or (2) referral to the public sector for any reason (table 1). Ideal management was determined based on practices reported in exit interviews. Provider knowledge was assessed by management practices described in case 1 vignettes, with additional context gleaned from general TB and HIV knowledge questions contained in knowledge surveys. The know-do gap was calculated as the percentage difference between ideally managed patients in case 1 vignettes (what providers ‘knew’) and case 1 exit interviews (what providers ‘did’) among the same sample of GPs (n=49). Bivariate logistic regression was used to consider factors associated with ideal case management: city, provider gender, patient–provider gender concordance, duration in practice (above and below 10 years^16^), interaction time (min), daily patient load (above and below 25 (median)), consultation fee and number of medications dispensed or prescribed. The number of TB symptoms queried (duration of cough, presence of: fever, night sweats, loss of weight/appetite)^17^ and whether or not HIV status was queried were evaluated for TB management; mention of TB was considered for HIV management. To account for the numerous bivariate comparisons made, a conservative p value of 0.01 was used to determine significance. Multivariate logistic regression was run to assess potential confounding or effect modification of visit time and provider gender on management outcomes. An SP visit was considered ‘detected’ in follow-up phone surveys if any GP suspected one or more fake patients who (1) reported cough or other TB-like symptoms and/or (2) shared a diagnostic laboratory report consistent with TB and/or (3) disclosed a history of TB. An SP detection rate of less than 5% was deemed acceptable in accordance with prior SP studies.^18^ Statistical analyses were performed using Stata V.15.

### Ethics

Written, informed consent was sought and obtained from all study participants to receive up to three unannounced SPs with undisclosed symptoms over a period of 6 months, and to participate in intake, detection and (potentially) knowledge surveys. GPs were assured confidentiality, with only aggregate-level outcomes reported.

### Patient and public involvement

The study aligns with principles of quality of care and patient-centredness. However, participants were medical practitioners. No patients were involved in study recruitment or conduct. GPs in the study regions were invited to CME-accredited events in which aggregate study results were shared and discussed.

## Results

Of 385 eligible GPs approached for the study, 221 (57%) consented (see online supplemental figure 1). Overall, 511 SP visits were completed over an 8-month period: 96 GPs participated in 220 SP visits (case 1: 95, case 2: 50, case 3: 75) from August to November 2018 in Durban; 116 GPs participated in 291 SP visits (case 1: 107, case 2: 107, case 3: 77) from April to July 2019 in Cape Town. Nine enrolled GPs were unavailable for visits. Participating GPs had a Bachelor of Medicine and Bachelor of Surgery (MBChB) degree, the South African and UK equivalent to a Doctor of Medicine (MD) degree before residency training. About one-quarter (26%) identified as women (table 2). The average consultation fee was ZAR320 (~US$22), range ZAR100–500. Average visit time was 9.6 min for typical TB and previous TB (cases 1 and 3) (95% CI 9.0 to 10.2) and 11.3 min for confirmed TB (case 2) (95% CI 9.8 to 12.5), p=0.02. One hundred and fifty-eight GPs were reached for detection surveys representing 387 visits (76%), among which, SPs were detected in nine (2.3%). On average, 3.1 medications were dispensed or prescribed per visit (SD=1.6, range 0–8), including at least one antibiotic in 76% of visits (95% CI 72% to 80%).

10.1136/bmjgh-2021-005250.supp1 Supplementary data

**Table 2:** Provider characteristics

Characteristic	Overall n=212	Durban n=96	Cape Town n=116
Sex, n (%)			
Male	156 (73.6)	82 (85.4)	74 (63.8)
Female	56 (26.4)	14 (14.6)	42 (36.2)
Area, n (%)			
City	46 (21.7)	43 (44.8)	3 (2.6)
Township	49 (23.1)	41 (42.7)	8 (6.9)
Suburb	117 (55.2)	12 (12.5)	105 (90.5)
Years in practice, median (IQR)	24.5 (15–35.5)	24 (15–33)	25 (14–38)
Daily patient load, median (IQR)	25 (15–30)	25 (15–33)	20 (15–30)
Consult fee in ZAR, median (IQR)	320 (280–360)	300 (250–350)	350 (300–380)

### Ideal management

**Figure 1:**
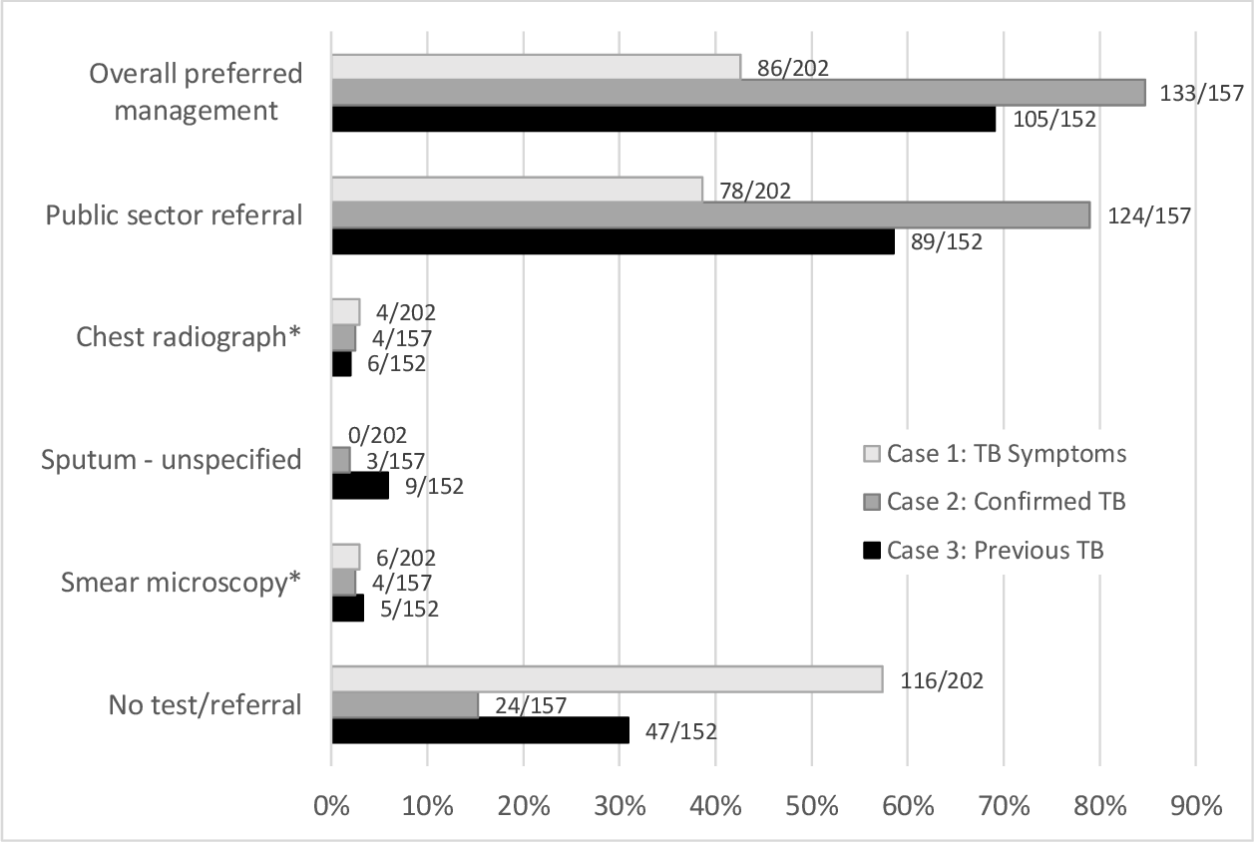
TB management by case presentation. *One case 1, four case 2s and three case 3s were referred for both smear microscopy and chest radiograph. TB, tuberculosis.

Ideal TB management was recorded in 63% of visits (95% CI 59% to 68%), among which 90% resulted in referral to the public sector. TB was specified in 79% of public sector referrals (95% CI 75% to 84%). Private test referrals (n=21) included smear microscopy (38%), chest X-ray (29%) and smear microscopy plus X-ray (33%). There were no referrals for GeneXpert. GPs requested sputum specimens in an additional 12 visits with TB test unspecified. Case 2 visits (confirmed TB) were most likely to be managed ideally: 85% (95% CI 78% to 90%), followed by case 3 (previous TB): 69% (95% CI 61% to 76%) and case 1 (typical TB): 43% (95% CI 36% to 50%), p<0.0001 (See figure 1).

Ideal HIV management occurred in 59% of visits (95% CI 54% to 63%), including 13% in which GPs offered an on-site HIV test, including CD4 or viral load testing for SPs who reported being HIV positive (95% CI 10% to 16%). HIV-related testing or treatment was specified in 39% of referrals to the public sector (95% CI 34% to 45%), and 1.8% of visits resulted in referral for private HIV-related tests (95% CI 0.8% to 3.3%). As with TB, HIV was most likely to be managed ideally among those presenting with confirmed TB: 80% (95% CI 52% to 68%) and least likely for those presenting with typical TB symptoms only: 41% (95% CI 34% to 48%), while those presenting with previous TB fell in between: 61% (95% CI 73% to 86%), see figure 1. HIV status was queried in 35% (95% CI 31% to 39%) of visits: 24% (95% CI 18% to 30%) of typical TB, 41% (95% CI 34% to 50%) of confirmed TB and 42% (95% CI 34% to 50%) of previous TB presentations (p<0.001).

### Factors associated with ideal management

The overall odds of ideal TB management in visits were higher among GPs who were women (OR=3.2, 95% CI 2.0 to 5.1), spent more time with SPs (OR=1.1 per additional minute, 95% CI 1.1 to 1.2), discussed HIV in the visit (OR=11.2, 95% CI 6.4 to 19.6) or practiced in Cape Town (OR=2.2, 95% CI 1.5 to 3.1) (table 3). Longer visit times were positively associated with inquiring about three or more common TB symptoms (duration of cough, night sweats, fever and weight loss) (OR=1.2 per minute, 95% CI 1.1 to 1.3) and inversely associated with dispensing more than three medications (OR=0.95 per minute, 95% CI 0.92 to 0.98). The overall odds of ideal HIV management in visits were similarly higher among GPs who were women (OR=3.2, 95% CI 2.0 to 5.1), spent more time with SPs (OR=1.1 per additional minute, 95% CI 1.1 to 1.2), discussed TB in the visit (OR_TB_=21.6, 95% CI 10.9 to 42.9) or practiced in Cape Town (2.2, 95% CI 1.5 to 3.1) (table 4).

**Table 3:** Bivariate analysis of factors associated with ideal TB management

	Overall OR (95% CI)	Case 1: typical TB OR (95% CI)	Case 2: confirmed TB OR (95% CI)	Case 3: previous TB OR (95% CI)
Cape Town	2.17 (1.50 to 3.13)*	2.37 (1.43 to 4.23)*	1.66 (0.68 to 4.05)	1.82 (0.90 to 3.66)
Female provider	3.18 (1.98 to 5.11)*	3.34 (1.73 to 6.42)*	11.76 (1.54 to 89.92)	2.86 (1.10 to 7.43)
Consult time (per additional minute)	1.12 (1.08 to 1.17)*	1.16 (1.09 to 1.23)*	1.11 (1.01 to 1.23)	1.10 (1.02 to 1.18)*
Mentioned HIV in visit	11.23 (6.43 to 19.59)*	21.08 (8.36 to 53.14)*	4.24 (1.37 to 13.07)*	8.76 (3.43 to 22.42)*
No. of medications dispensed/prescribed	0.60 (0.52 to 0.69)*	0.73 (0.58 to 0.93)*	0.57 (0.41 to 0.78)*	0.70 (0.54 to 0.90)*
No. of common symptoms queried†	1.99 (1.70 to 2.33)*	2.72 (2.00 to 3.71)*	2.11 (1.46 to 3.06)*	1.75 (1.34 to 2.28)*

*p≤0.01.

†Common symptom questions: duration of cough, presence of: fever, night sweats, weight loss.

TB, tuberculosis.

**Table 4:** Bivariate analysis of factors associated with ideal HIV management

	Overall OR (95% CI)	Case 1: typical TB OR (95% CI)	Case 2: confirmed TB OR (95% CI)	Case 3: previous TB OR (95% CI)
Cape Town	2.38 (1.66 to 3.42)*	2.32 (1.30 to 4.13)*	1.92 (0.87 to 4.27)	2.29 (1.17 to 4.45)*
Female provider	3.48 (2.20 to 5.50)*	2.63 (1.38 to 4.99)*	8.15 (1.86 to 35.71)*	5.87 (2.13 to 16.12)*
Consult time (per minute)	1.11 (1.07 to 1.16)*	1.15 (1.08 to 1.22)*	1.07 (0.99 to 1.15)	1.11 (1.04 to 1.18)*
Mentioned TB in visit	21.57 (10.85 to 42.87)*	13.05 (5.28 to 32.27)*	64.95 (7.96 to 529.73)*	13.75 (3.85 to 49.08)*
No. of medications dispensed/prescribed	0.61 (0.53 to 0.69)*	0.80 (0.63 to 1.01)	0.56 (0.42 to 0.75)*	0.65 (0.50 to 0.83)*

*p≤0.01.

TB, tuberculosis.

On average, female GPs spent an average of 3.3 min longer with patients compared with their male counterparts (95% CI 2.4 to 4.3, p<0.0001), thus we ran multivariate analyses to consider the combined effects of provider gender and visit time on ideal management. Multivariate results indicated the odds of ideal TB and HIV management were, respectively, 2.6 (95% CI 1.6 to 4.2) and 2.9 (95% CI 1.8 to 4.6) times higher for female versus male providers, and 1.1 (95% CI 1.1 to 1.2) and 1.1 (95% CI 1.06 to 1.14) times higher per minute of consultation time, with p<0.0001 for all comparisons.

### Know-do gap

Overall, 49 GPs completed a knowledge survey, wherein 80% (95% CI 66% to 90%) reported ideal management for TB and 55% (95% CI 40% to 69%) for HIV (p<0.001) in response to a case vignette reflecting typical TB symptoms (case 1). This compared with 43% ideal management for TB (95% CI 29% to 58%) and 37% for HIV (95% CI 23% to 52%) (p=0.37) in typical TB (case 1) visits involving the same set of GPs. Accordingly, the know-do gaps of 37% for TB (95% CI 19% to 55%) and 18% for HIV (95% CI −1% to 38%) were evident.

### Other factors explaining ideal management

Key determinants of GP clinical decisions emerged within knowledge surveys that were not measured during SP visits. Among surveyed GPs, a minority of providers (20%, 95% CI 10% to 34%) indicated they would consider TB in persons with sickly appearance, haemoptysis or known TB contact, while 49% (95% CI 34% to 64%) and 39% (95% CI 25% to 54%) said they would consider HIV in persons with apparent weight loss/emaciation or sickly appearance/frequent illness. These attributes were not made explicit by SPs’ physical appearance or case histories. Further, 43% of GPs (95% CI 29% to 58%) indicated they would manage cash-paying patients, representative of all our SPs, differently from insured patients; that is, they would refer the former to the public sector whereas the latter for private testing. Finally, 84% of surveyed GPs reported smear microscopy (95% CI 70% to 93%) and 61% reported chest X-rays (95% CI 46% to 75%) as the preferred first test(s) for TB; six GPs revealed within open-ended responses that this preference was due to the lower test cost (US$3) when compared with the cost of more effective tests such as GeneXpert (US$26–70) and culture (US$12).

GP estimates of HIV prevalence among clientele varied greatly and differed by site. In Durban the mean estimate was 18%, median 11% and range 1%–80%; while in Cape Town the mean estimate was 8%, median 5% and range 0%–25%.

## Discussion

This is the first study using SPs to assess quality of care for TB in South Africa’s private sector, which provides primary healthcare services to about a third of the population.^4^ Findings revealed novel insights and opportunities for improvement, particularly with regard to TB symptom and diagnostic management, and uniquely about HIV management. While TB quality improvement (QI) programmes are ongoing in South Africa’s public sector,^19^ these QI projects currently do not cover the private health sector.

Study GPs performed significantly better when TB was explicitly part of the case presentation, with 85% of confirmed TB (case 2) and 69% of previous TB (case 3) presentations resulting in a referral to the public sector or for TB testing compared with 43% of typical TB (case 1) presentations where the diagnosis was unclear. In this study, GPs provided superior quality of TB care compared with private providers receiving similar case presentations in other high-burden settings including India and Kenya,^11–13^ where typical TB symptoms (akin to case 1) were ideally managed in 12%–36% of visits. However, our study GPs did not perform as well as clinicians in the public sector in South Africa,^10^ Kenya^13^ and tertiary centres in China,^20^ where typical TB symptoms were ideally managed in 79%–90% of visits.

While GPs performed reasonably well in case 1 vignettes (80%), the same GPs scored 37% lower in actual practice. The absence of a case history more suggestive of TB or obvious signs of wasting may explain some of this difference; however, these signs are neither likely nor relevant in early stages of TB when timely diagnosis and treatment is crucial to reduce morbidity and community transmission. Presentations of TB are also more likely to be atypical in populations with a high prevalence of HIV.^21^

Perceived patient costs may have played a role in GP decisions. TB was specified in 79% of visits that resulted in referral to the public sector, where GeneXpert testing is provided at no cost to patients. A good majority of GPs reported smear microscopy and chest X-rays as preferred first tests if referring patients within the private sector, both of which are markedly cheaper than GeneXpert with comparatively less accuracy.^22 23^ Moreover, a substantial proportion of GPs reported managing cash-paying patients distinctively from insured patients, with greater consideration of patient costs for the former. Combined with the high proportion of public sector referrals as compared with similar studies,^11–13^ these findings suggest an opportunity to engage private providers in South Africa in ways that favour cost-savings for their patients without compromising test accuracy.

Observed management of HIV in this study followed a similar pattern to TB, mostly driven by public sector referral. To date, no SP studies have assessed quality of HIV care among clinicians in the private sector, hence findings from this study may provide a baseline for future inquiries. Given the high prevalence of HIV, South African guidelines recommends HIV screening for all patients at every health visit,^24^ but the HIV status of SPs was queried in only a third of visits (35%), and least among SPs presenting with typical TB symptoms (24%). This is substantially lower than the rate of inquiry in a similar SP study in South Africa’s public sector (49%),^10^ and points to a lost opportunity for private providers to deliver high quality HIV care among a high-risk population. The narrower know-do gap for HIV management, 18% versus 37% for TB, is not indicative of better HIV management compared with TB, but of lower theoretical performance in vignettes more in line with actual practice.

Knowledge survey responses may partly explain suboptimal vignette and visit performance in relation to HIV screening and management. GP estimates of HIV prevalence among clientele varied greatly from 1% to 80% in Durban and 0%–25% in Cape Town, while actual prevalence is reported at 17% and 10%, respectively.^25^ Although prevalence may differ by community, it is unlikely that sampled GPs would find little or no HIV among patients. Knowledge survey responses suggest some GPs may be seeking tangible signs of wasting, sickly appearance or chronic infections before considering HIV. A systematic review of barriers to offering HIV tests to patients has shown that interpersonal barriers such as fear of offending patients, cultural barriers between providers and their patients and concerns about delivering a positive result can also prevent clinicians from assessing HIV within routine clinical visits.^26^ Research is thus needed to determine strategies to empower providers to routinely query and test for HIV in the South African context.

Several factors underpinned ideal case management. For every additional minute spent interacting with SPs, the odds of ideal TB management increased by 12%. Longer visits were also associated with more thorough symptom inquiry (OR=1.2) and ideal HIV management (OR=1.1). Moreover, providers who spent less time with patients were more likely to prescribe more medication (OR=1.1), including antibiotics. These findings are consistent with a systematic review which found that longer GP consultation times improve general elements of care.^27^ Lastly, female providers showed higher rates of ideal TB and HIV management. A gender difference remained despite controlling for length of visit, suggesting other factors related to gender play a role in ideally managing TB and HIV. This finding is echoed in a recent SP study in Nigeria in which female providers had better TB management outcomes, regardless of SP gender,^28^ but differs from a study in India which found no difference in TB management outcome by provider or SP gender.^29^

Private sector engagement is identified as a priority in South Africa’s National Strategic Plan on HIV, TB and STIs.^30^ While a National Health Insurance scheme that would pool funds to provide equal healthcare access regardless of socioeconomic status is in the pipeline,^31^ currently private sector engagement in TB and HIV is largely limited to voluntary CME seminars offered through university and clinical organisations. While the national TB programme has sought to add TB treatment to the list of chronic medications that South Africans can access through public stock at registered access points, including private GPs, this service is yet to become available. As our data suggest, more needs to be done to improve opportunities for GPs to connect patients to appropriate TB diagnostics and treatment to minimise losses in the care cascade. Such initiatives could include connecting patients of private GPs to affordable testing through partnerships with National Health Laboratory Services, leveraging the accomplishments of COVID-19 testing and reporting in both public and private sectors, and expansion of minimum prescribed benefits provided through medical insurers to include TB testing by GeneXpert and HIV testing by ELISA, viral load and CD4 cell count through private laboratories. Additionally, routine quality assessments for TB and HIV care in the private sector will help to identify successful interventions and areas where improvement may still be required.

This study has some limitations. First, evaluations were based on a single visit, and may not be indicative of typical clinical practices; however, follow-up visits would have increased the risk of SP detection. Second, visits were not recorded and are therefore subject to recall bias. Exit interviews were facilitated immediately following visits and SPs were extensively trained to minimise this risk, but novel practices, accents and medical terminology in real-life visits may have posed challenges. Third, our consent process differed from most SP studies in which consent waivers were granted by ethics bodies.^10–12 20^ If GPs with greater clinical abilities or interest in evidence-based clinical practice were more likely to consent, we may have over-estimated ideal management practices, making direct comparison to other settings less accurate. Lastly, since our SPs did not appear sick or malnourished, this might have influenced provider judgements, an inherent limitation of the SP methodology. Further research is needed to understand the complexities that prevent private GPs from connecting patients to TB testing sooner, why short-term antibiotic use is so frequently used even when GPs have a high level of suspicion for TB, and how providers could be empowered to routinely test for HIV in the South African context.

## Conclusion

Private providers in South Africa ideally managed TB more often when a diagnosis or history of TB was implied. Management of HIV in the context of TB was less than optimal. Knowledge surveys suggested that cost implications for patients or an expectation of overt signs of illness, atypical of early-stage disease, may delay ideal TB or HIV management. Factors that improved management practices included longer visit times, female providers, typical symptom inquiry and city. Dispensing of medications was inversely related to ideal management of both TB and HIV. Collectively, these findings could help expand QI programmes to also cover the private health sector in the country.

## Data Availability

All data relevant to the study are de-identified and included in the article or uploaded as supplementary information.
