# Supplementary material for: Quality of care for tuberculosis and HIV in the private health sector: a cross-sectional, standardised patient study in South Africa
Source: BMJ Glob Health. 2021 May 14;6(5):e005250. doi: 10.1136/bmjgh-2021-005250 (PMC8127976; doi:10.1136/bmjgh-2021-005250)
Supplement: Supplementary data [file bmjgh-2021-005250supp001.pdf]

Supp 1: Consent Diagram

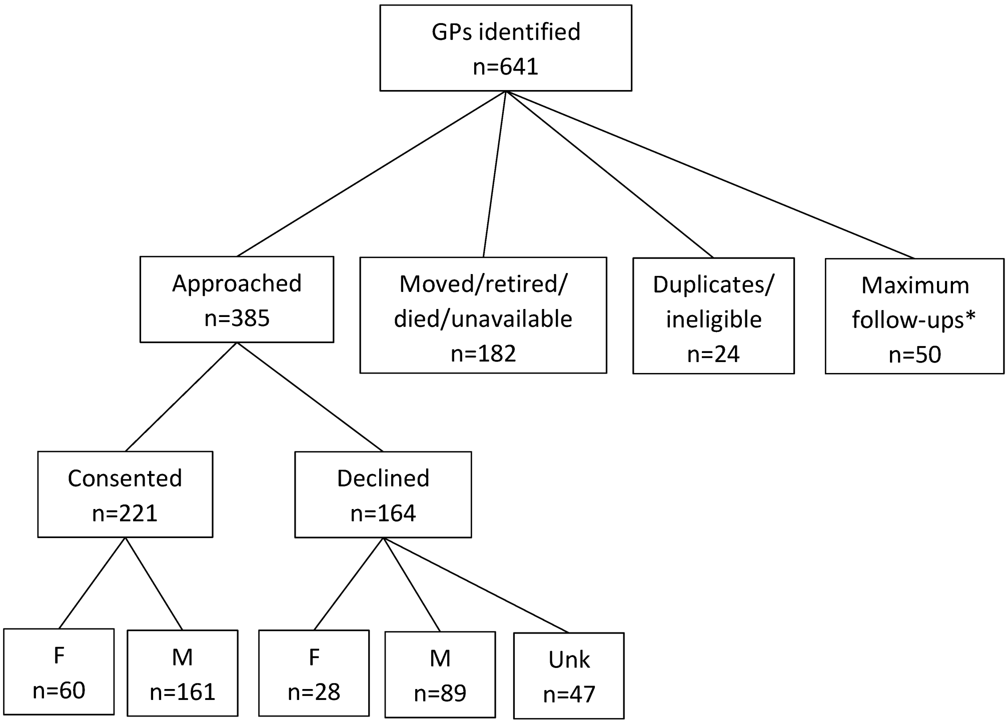

*GPs were approached a maximum of 5 times by recruitment staff
Abbreviations: GPs=general practitioners, Unk=unknown
